# Psychometric evaluation of the opiate dosage adequacy scale: reliability and convergent validity among non-European Spanish-speaking buprenorphine patients

**DOI:** 10.3389/fpsyt.2025.1644076

**Published:** 2025-11-14

**Authors:** Thairys Flores Ocasio, Nicole Melendez Rivera, Jorge Duconge, Raman Venkataramanan, Francisco González-Saiz, Darlene Santiago

**Affiliations:** 1 Department of Pharmaceutical Sciences, School of Pharmacy, University of Puerto Rico, San Juan, Puerto Rico; 2 Biostatistics, Epidemiology and Research Design Core (BERD), The Hispanic Alliance for Clinical and Translational Research, University of Puerto Rico, San Juan, Puerto Rico; 3 Pharmaceutical Sciences and Pathology Department, School of Pharmacy and Medicine, University of Pittsburgh, Pittsburgh, PA, United States; 4 Unidad de Hospitalizacion de Salud Mental, Hospital Universitario de Jerez, Universidad de Cadiz, Cadiz, Spain

**Keywords:** opiate dosage adequacy scale, ODAS, opioid use disorder, buprenorphine, convergent validity, realiability

## 1 Introduction

The Opiate Dosage Adequacy Scale (ODAS) is a semi-structured clinical interview designed for estimating the adequacy of methadone or buprenorphine doses for patients with opioid use disorder (OUD) (1, 2). ODAS’s effectiveness relies on its proven ability to estimate the adequacy of a given dose based on multiple clinical parameters including continuous heroin use, cravings, narcotic blockage, objective and subjective withdrawal symptoms, and signs of overmedication (3, 4). The ODAS was originally developed and validated with Spanish-speaking European patients receiving medication for OUD (5, 6). The original validation demonstrated good reliability and construct validity, with Cronbach’s alpha coefficients ranging between 0.76 (methadone patients) and 0.92 (buprenorphine patients), with coefficients for each of the domains ranging between 0.81 and 0.93 (1, 2). ODAS scores were correlated with clinician assessments of treatment sufficiency and with patient self-reported outcomes, underscoring its clinical relevance in opioid maintenance therapy. Subsequent studies confirmed that ODAS-based dose classification is aligned with longitudinal treatment outcomes, reinforcing its clinical applicability in opioid maintenance therapy, although no Cronbach’s alpha was reported (4). Beyond European settings, the ODAS has been validated for cross-cultural use. A Persian adaptation of the ODAS demonstrated comparable psychometric properties, confirming its internal consistency (Cronbach’s α > 0.70) and convergent validity with other addiction severity measures (e.g., the Severity of Dependence Scale, SDS) (7). These findings suggest that the ODAS is a robust and adaptable instrument, capable of assessing opioid dose adequacy across diverse treatment populations. However, its applicability to other non-European Spanish-speaking populations remains unstudied.

Given the complexities of OUD pharmacotherapy, the ODAS provides a standardized approach for dose assessment that integrates both clinical symptoms and patient-reported outcomes. Previous validation studies have demonstrated its strong reliability and validity for determining whether a patient’s medication dose is sufficient to maintain clinical stabilization (2, 4). Despite the growing burden of opioid use disorder among Hispanic and Spanish-speaking individuals in the United States and across Latin America, few validated tools exist for guiding opioid dose adjustments in a linguistically and culturally relevant manner (8, 9). Given the clinical importance of ensuring appropriate buprenorphine dosing in opioid maintenance therapy, there is a critical need for validated instruments that are not only clinically sound but also tailored to the language and cultural context of diverse populations. Expanding the validation of the ODAS beyond its European origins to include Spanish-speaking populations in the Americas can contribute significantly to addressing treatment disparities and supporting individualized care strategies in these communities. Importantly, psychometric properties of clinical tools often vary across cultural and clinical contexts; hence, the ODAS measures developed in one population may not fully capture treatment adequacy in another. This gap is particularly important for Puerto Rican and other Hispanic subgroups who are disproportionally affected by opioid use disorder and who may experience distinct patterns of treatment access and retention. Validating the ODAS in these populations is therefore critical to ensure its reliability, validity, and applicability in these clinical settings, and to promote equitable assessments of treatment adequacy.

In this study, we aim to evaluate further the ODAS’s psychometric properties and its applicability to Spanish-speaking patients outside of Europe receiving buprenorphine OUD therapy evaluating the scale’s internal consistency (Cronbach’s alpha coefficient) and convergent associations with clinical stabilization variables such as severity of dependence (using the Severity of Dependence Scale [SDS]) and illicit drug use (2). We also aimed to examine the associations with patients’ buprenorphine levels (1).

## 2 Materials and methods

The institutional review board of the University of Puerto Rico Medical Sciences Campus approved the study before the recruitment started (#B1080320).

### 2.1 Participants and inclusion/exclusion criteria

This is a cross-sectional study that recruited 111 subjects. The inclusion criteria were ≥ 21 years old, being a patient under buprenorphine treatment for OUD for at least 3 months and taking a maintenance dose, and not having any changes in said buprenorphine dose during the week prior to participation in the study. The following patients were excluded: those with a social or mental incapacity (i.e., inability to understand or answer the questions on the ODAS), those who had started buprenorphine treatment less than 3 months prior to the interview, and those who were pregnant.

### 2.2 Recruitment sites and process

Two clinics in Puerto Rico with similar patient demographics were used to recruit participants. Both are funded by the United States Department of Health and Human Services Health Resources and Services Administration to provide care for economically disadvantaged people; at these clinics, patients receive whatever services they need, even if they cannot afford them. One clinic is located near the San Juan metropolitan area, and the other is located near the central east side of the island; their names are being kept confidential to maintain the anonymity of the recruited subjects. Both clinics primarily offer Spanish services to patients with OUD who speak Spanish. After the clinic assessed the inclusion and exclusion criteria, the study team proceeded to obtain written consent. Subjects were interviewed once to collect both data (ODAS and SDS questionnaire) and a blood sample; the researchers then collected additional information from the participant’s medical records. All the interviews and patient interactions took place face-to-face. The opiate dose adequacy scale (ODAS) and all clinical stabilization variable responses were collected during a routine appointment in a single interview based on patients’ status in the past 7 days.

### 2.3 Opiate dosage adequacy scale

The ODAS instrument is a Spanish-validated scale used to estimate the adequacy of buprenorphine dosing (1, 2). It involves a guided clinical interview in which the interviewer asks the patient specific questions about their experiences within the last week of the use of medication for OUD. Depending on the responses provided, the interviewer will either continue with the order or skip to the next questions. The interviewer will collect the responses from the patient. The interview consists of 10 questions, and it employs a multidimensional approach that assesses frequency of heroin use (Item 1) and its effect (narcotic blockade or crossed tolerance; Item 2), objective/subjective opioid withdrawal symptoms frequency and intensity of such symptoms (Items 3 and 4), heroin cravings frequency and intensity (Item 5), and overmedication frequency and intensity of symptoms (Item 6). Questions that measure symptom frequency are coded with Likert-type scale values (none of the last seven days, two or three of the last seven days, five or six of the last seven days, one or two times a day every day, three or more times a day every day; scored 5, 4, 3, 2, 1). Questions that measure symptom intensity are coded on a visual analogue scale 1-5 (no effect, 1; the effect was extremely intense, 5). The quantitative total ODAS score is calculated by the interviewer as the sum of all item scores, ranging from 6 to 30. If a patient reports not using illicit opioids, does not experience withdrawal symptoms or heroin cravings (narcotic blockade or crossed tolerance), does not experience signs or symptoms of overmedication, then a patient is considered to be “adequately dosed; ODAS score of 30.” (2) Higher scores indicate greater dose adequacy, while lower scores suggest dose inadequacy; however, the optimal cut-off points for determining dose adequacy are limited (1). This instrument also allows for dose adequacy to be interpreted qualitatively (yes or no) by assigning an adequate dose (yes) to patients who score 4 or 5 in all six items. Those who do not meet this condition are classified as patients with inadequate doses (no) (1).

For the purpose of this analysis, the ODAS qualitative dose adequacy condition was used to determine if a participant was considered adequately dosed (yes) or not (no). (1) The quantitative total score of dose adequacy of ODAS was used to assess the convergent validity with clinical variables such as severity of dependence, toxicology (illicit opioid use) and plasma levels of buprenorphine, and to estimate a clinically meaningful ODAS threshold (cut-off score) of dose adequacy.

### 2.4 Clinical stabilization variables

#### 2.4.1 Severity of dependence scale

The Severity of Dependence Scale (SDS) is a 5-item scale that assesses substance-dependence severity (including cravings and withdrawal) over the previous 12 months. The responses to each item are coded on a Likert-type scale, and the total SDS score can range from 0 to 15 points, with a higher score indicating a greater degree of drug dependence. The SDS was originally developed by Gossop et al. (10) (10) and later translated into and adapted to Spanish (11).

#### 2.4.2 Illicit drug use (toxicology results)

On the day of the study, the data on each participant’s electronic health record pertaining to urine toxicology results for heroin, cocaine, and unprescribed use of benzodiazepines, opioids, and/or fentanyl were used as a measure of illicit drug use (by means of urine drug testing kits).

### 2.5 Buprenorphine and metabolite plasma analysis

A single 5 mL blood sample using lavender/purple-colored EDTA vacutainer tubes was collected. Whole blood samples were centrifuged (3000 rpm; 10 minutes) to obtain plasma (supernatant). Plasma was stored at -80 °C in cryogenic tubes for the subsequent analytical analysis. Protein precipitation of plasma samples combined with ultra-high performance liquid-chromatography-mass spectroscopy was used to quantitate buprenorphine and three of its metabolites (norbuprenorphine, buprenorphine-3-glucuronide, and norbuprenorphine-3-glucuronide) (12).

### 2.6 Statistical analyses

Descriptive statistics were used to assess the distribution of sociodemographic characteristics (e.g., sex, age, education) among the sample of participants, these were presented with their respective frequencies and percentages. Mann-Whitney and Chi-squared tests were employed, as appropriate, to identify potential associations between opioid dose adequacy in its categorical form (yes/no) and each of the individual symptom frequency item scores (heroin use, narcotic blockade, subjective/objective symptoms, cravings, and overmedication; Likert-type scale values). Two-sided tests were used, and the mean, standard deviation, and respective percentiles were reported. To observe the correlation between the ODAS continuous score and the different clinical stabilization measures (SDS, illicit substance use, buprenorphine dose and plasma levels), Spearman and Pearson correlations were carried out. The internal consistency of the ODAS quantitative adequacy (6 items) was estimated using Cronbach’s alpha coefficient (mean standardized). (13) All statistical analyses were conducted using STATA/MP Parallel Edition 17.0. software (14).

A receiver operating characteristic (ROC) curve analysis was conducted to estimate an optimal ODAS cut-off for classifying dose adequacy (Python 3.11 and the Scikit-learn library v1.3). Using dose adequacy (yes or no) as the classification outcome (see section 2.2), cut-off values were determined based on the maximum Youden index of the area under the ROC curve (AUC) (15).

A sample size calculation showed that at least 96 participants are needed to have 80% power to detect a medium effect size at a two-sided significance level of α = 0.05, ensuring the study is adequately powered for psychometric validation.

## 3 Results

### 3.1 Demographics, past medical history, and pharmacological treatment of sample

In our sample, 101 out of 111 (90.99%) of the participants were men, 44% (N=39.64) had a high school degree or more (55% had high school as its highest degree) and 70% (N=63.06) were employed (**Table 1**). The mean age of the sample was 43 ( ± 10) years. All the patients were enrolled in the health care program provided by the government of Puerto Rico. Participants self-identified as Hispanic or Latino (72%), White (2%), or other (36%).

**Table 1:** Demographics of recruited subjects (n=111).

Variable	Overall n (%)
Age	
Mean ± SD	43 ± 10
Median (P25 – P75)	41 (37, 51)
Sex	
Male	101 (90.99)
Female	10 (9.01)
Education Groups	
Less than High School	67 (60.36)
High School or more	44 (39.64)
Highest Education Attained*	
Elementary School	3 (3.00)
Middle School	9 (9.00)
High School	55 (55.00)
Technical/associate degree	15 (15.00)
College/bachelor’s degree	18 (18.00)
Employment	
Employed	70 (63.06)
Not Employed	41 (36.94)

*Variable has 11 missing values.

### 3.2 Opiate dosage adequacy scale

Qualitative adequacy: About 61 subjects (54.9%) of our sample were receiving an adequate dose of buprenorphine with a mean score of 29.79, as determined by the ODAS; 50 subjects had inadequate doses (45.0%; mean ODAS score of 23.60) (**Table 2**). Statistical analyses revealed significant differences among the dose adequacy groups for each item on the ODAS scale, as well as the total ODAS score (P-value < 0.001).

**Table 2:** ODAS item scores comparison with determined dose adequacy (n=111).

ODAS item	Inadequate dose (N=50; 45.0%)	Adequate dose (N=61; 54.9%)	P-value^a^
Continuous heroin use			
Mean ± SD	4.22 ± 1.22	4.98 ± 0.13	<0.001*
Median (P25, P75)	5 (4,5)	5 (5, 5)	
Cravings			
Mean ± SD	4.16 ± 1.35	4.98 ± 0.13	<0.001*
Median (P25, P75)	5 (4, 5)	5 (5, 5)	
Narcotic blockade			
Mean ± SD	3.58 ± 1.50	4.98 ± 0.13	<0.001*
Median (P25, P75)	4 (2, 5)	5 (5, 5)	
Objective withdrawal symptoms			
Mean ± SD	3.00 ± 1.48	4.90 ± 0.30	<0.001*
Median (P25, P75)	3 (2, 5)	5 (5, 5)	
Subjective withdrawal symptoms			
Mean ± SD	4.24 ± 1.29	4.95 ± 0.22	<0.001*
Median (P25, P75)	5 (3, 5)	5 (5, 5)	
Overmedication			
Mean ± SD	4.4 ± 1.18	4.98 ± 0.13	<0.001*
Median (P25, P75)	5 (5, 5)	5 (5, 5)	
Total ODAS Score			
Mean ± SD	23.60 ± 3.10	29.79 ± 0.61	<0.001*
Median (P25, P75)	24 (21, 26)	30 (30, 30)	

a P-values were calculated using Mann-Whitney or Chi-square, as appropriate.

*Results were statistically significant (p < 0.05).

In this sample, the Cronbach’s alpha coefficient was 0.615, thus supporting a moderate internal consistency of the quantitative dose adequacy (16).

The ROC analysis revealed that ODAS scores ≥ 29 were highly predictive of dose adequacy, with an AUC of 0.996, demonstrating strong discriminative power.

### 3.3 Opiate dosage adequacy scale validity

#### 3.3.1 ODAS adequacy scores, clinical stabilization measures, and buprenorphine plasma concentrations

**Table 3** illustrates the mean and median values of the clinical stabilization measures (Severity of Dependence, illicit substance use, and buprenorphine dose and plasma levels) for the dose adequacy groups as qualitatively determined. **Table 3** also shows the correlation coefficients between these clinical stabilization measures and the quantitative ODAS score, as well as its p-value. Moderately strong correlations were found between the clinical stabilization measures and dose adequacy as determined by the ODAS. The severity of dependence, illicit drug use, and buprenorphine dose all resulted in being inversely proportional with the ODAS score (all statistically significant). This means that the greater the severity of dependence, the lower the ODAS score (less adequate). The same resulted for the use of illicit opioids; more frequently reported substance use resulted in lower ODAS scores, hence less probable adequate doses. Higher buprenorphine doses were associated with lower ODAS scores (less dose adequacy). Buprenorphine plasma levels were inversely correlated to the ODAS score, meaning that increasing plasma levels correlated with a higher probability of dose adequacy.

**Table 3:** ODAS adequacy scores and clinical stabilization measures (n=111).

Variable	Inadequate dose (N=50)	Adequate dose (N=61)	Correlation with ODAS score	
			Coefficient	P-value^e^
Severity of Dependence (SDS)^a^			*r_s_*=-0.41	p < 0.001*
Mean ± SD	6.18 ± 3.47	3.85 ± 3.69		
Median (P25, P75)	7.00 (3.00, 9.00)	3.00 (1.00, 6.50)		
Illicit Substance Use^b^			*r*=-0.29	*p < 0.002* ^f^ *
Yes	42 (52.50)	38 (47.50)		
No	6 (21.43)	22 (78.57)		
Buprenorphine dose (mg)^c^			*r_s_*=-0.33	*p < 0.001* *
Mean ± SD	17.92 ± 6.22	14.85 ± 6.30		
Median (P25, P75)	16.00 (16.00, 24.00)	12.00 (8.00, 24.00)		
Buprenorphine plasma (ng/mL)			*r_s_* = 0.18	*p = 0.057*
Mean ± SD	2.44 ± 2.48	3.36 ± 3.04		
Median (P25, P75)	1.60 (0.57, 2.74)	2.87 (1.11, 4.10)		

a Variable has 2 missing values.

b Variable has 3 missing values. Results obtained from toxicology results in HER.

c Variable has 4 missing values.

e Statistical analysis was carried out using Spearman’s Correlation unless otherwise specified.

f Statistical analysis was carried out using Pearson Correlation.

*Results were statistically significant (p < 0.05).

### 3.4 Plasma-to-dose ratio by dosing frequency

**Figure 1:**
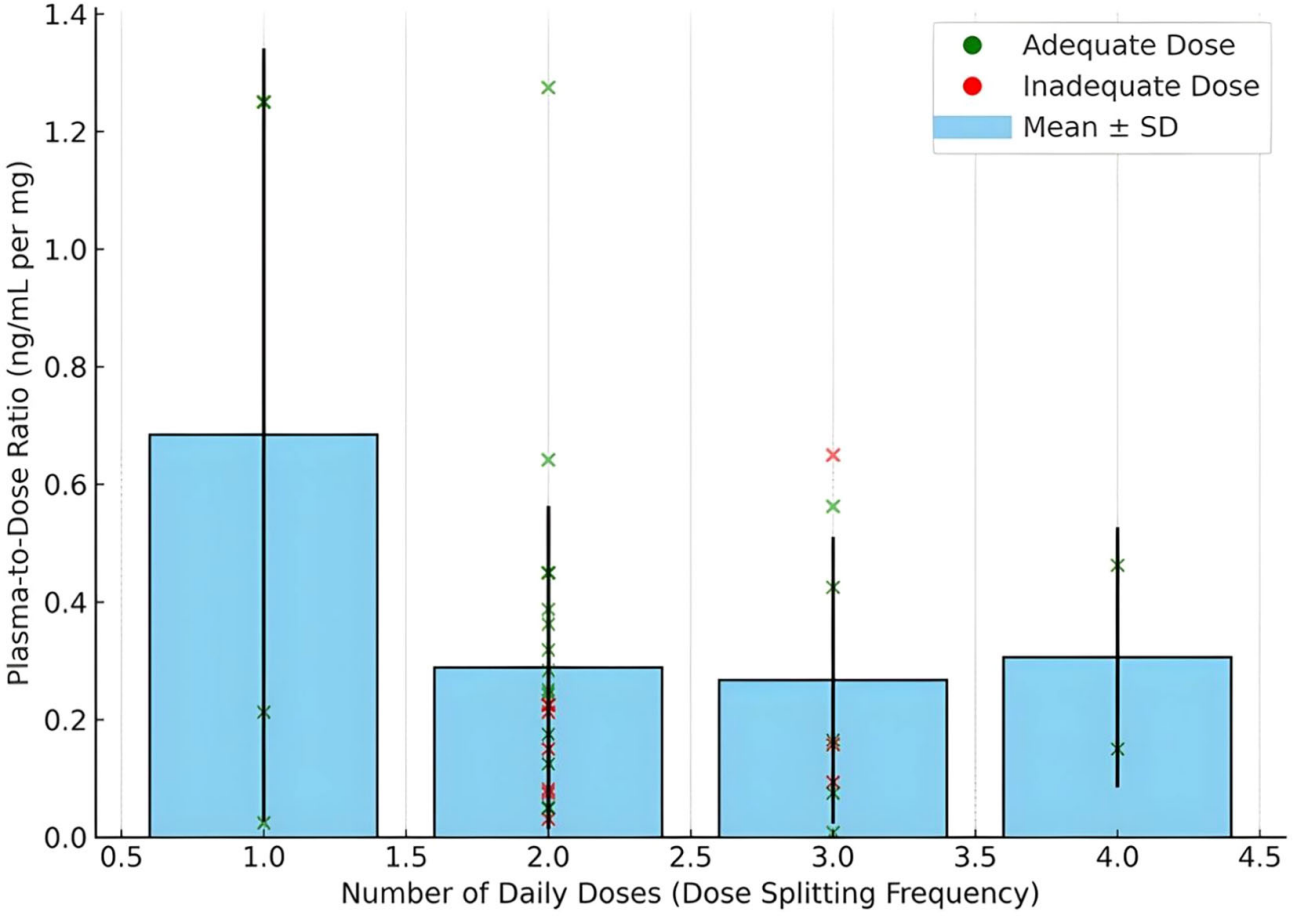
Plasma concentration per mg of Buprenorphine (daily dose) by dosing frequency of recruited subjects as determined by ODAS. Bars represent mean values with standard deviation error bars. Individual dots indicate observed plasma/dose values per participant. The data illustrate variability in plasma exposure efficiency across dose splitting patterns.

To further explore the observed variability in dose, dose adequacy and plasma levels, we examined the mean plasma concentration per mg of buprenorphine (plasma/dose ratio) across dosing frequencies. As shown in **Figure 1**, participants who split their daily dose more frequently did not consistently achieve higher plasma exposure per mg, and variability was notable across all frequency groups. Individual data points and standard deviations illustrate this heterogeneity in exposure efficiency.

## 4 Discussion

The results reported herein support the use of the Opiate Dose Adequacy Scale (ODAS) in Spanish-speaking, non-European individuals maintained on buprenorphine. Our study subjects closely matched the demographic profiles, including age, sex, education, and employment distributions, of those in previously reported ODAS convergent validity studies conducted in Europe (2).

The dose-adequacy distribution of recruited subjects (54.9% were adequately dosed; mean total score: 29.79 ± 0.61) was lower than that reported for Europeans (73.3%; mean total score: 28.58 ± 2.55); more patients had an inadequate dose in this study (2). Our results demonstrated the ODAS’s capability to discriminate between adequately and inadequately dosed individuals, as evidenced by statistically different ODAS total scores between groups. Item 4, *objective withdrawal symptoms*, had the lowest scores in both groups (which was also the case for the European subjects). This item can then be an indicator of dose inadequacy (subtotal score < 4) in this population; a major cause for dose inadequacy. The optimal ODAS cut-off score for this group has been identified as 29. Patients with scores greater than 29 are significantly more likely to receive an adequate dose, while those with scores less than 29 tend to be inadequately dosed. Further studies are essential to validate this cut-off value for a clear classification of dose adequacy within this population, particularly focusing on patients’ objective withdrawal symptoms (item 4), as these symptoms can significantly impact dose adequacy. These results, consistent with previous findings, have demonstrated that the ODAS can be used to determine dose adequacy in a group of non-European Spanish-speaking patients with OUD and, as such, can be used as a clinical assessment for providers to identify patients that require a dose adjustment and improve maintenance treatment effectiveness (1, 2, 4). Further implementation studies should be conducted to test this intervention properly.

While the internal consistency of the ODAS in this sample was moderate (Cronbach’s α = 0.615), we acknowledge that Cronbach’s alpha has certain limitations when applied to Likert-type scales. Specifically, it assumes tau-equivalence and continuous measurement, which may not fully reflect the reliability of ordinal, symptom-based responses. Future analyses should consider alternative indices such as ordinal alpha or McDonald’s omega, which are better suited for ordinal data and can provide more robust estimates of internal consistency (17). Additionally, inter-rater reliability (IRR) was not assessed in this study because all interviews were conducted by a single trained researcher. While this minimized variability in scoring, it also limits our ability to assess consistency across different raters. Future studies should evaluate IRR through standardized methods (e.g., intraclass correlation or Cohen’s kappa), particularly given the semi-structured format of the ODAS, to ensure generalizability across clinical settings.

One important finding of this study was that dose adequacy was inversely proportional to the prescribed daily dose of buprenorphine; inadequately dosed patients had higher prescribed doses than those with adequate doses. Inadequately dosed patients in this study, having mean plasma values of 2.44 ng/mL (median 1.60), can explain their low treatment effectiveness (dose inadequacy) even though having higher doses than the adequately dosed group. Plasma levels of ≥ 3 ng/mL are required for a higher (> 80%) saturation of the opioid receptors and, therefore, treatment effectiveness (18). Patients in the adequate dose group had average plasma levels of 3.36 ng/mL. The reason for patients with inadequate doses having lower plasma levels than patients with adequate doses, even though taking relatively higher doses, may be explained by a combination of patient compliance regarding dosing schedules and pharmacological characteristics in this group.

To further explore the observed results between buprenorphine dose, plasma levels, and clinical dose adequacy, we examined the plasma-to-dose ratio (ng/mL per mg of buprenorphine) across different dose splitting frequencies. As illustrated in **Figure 1**, mean plasma-to-dose ratios varied across dosing patterns, with high inter-individual variability evident within each group. When stratified by dose adequacy, the plot shows that adequately dosed participants (green) generally achieved higher plasma exposure efficiency than inadequately dosed participants (red), even when using similar or lower total daily doses. This suggests that higher prescribed doses in the inadequate group did not translate into proportionally higher plasma concentrations, potentially due to suboptimal adherence, metabolic differences, or pharmacokinetic fluctuations associated with irregular dosing schedules.

Notably, the majority of participants in this sample reported dividing their daily buprenorphine dose into two to six doses per day, often following asymmetric or personalized patterns. These practices, while common, may lead to variability in trough plasma levels, particularly in the inadequate group. The data underscore the importance of considering pharmacokinetic efficiency, not just total dose, in assessing and optimizing buprenorphine treatment. Incorporating both clinical assessment tools like ODAS and objective pharmacokinetic data may offer a more complete understanding of dose adequacy in diverse treatment populations. Evidenced-based dosing schemes are an alternative, even more so in the current fentanyl era within the opioid crisis where higher (>24 mg) and varied dosing schemes are proposed as treatment effectiveness increase strategies (19, 20).

Building on these findings, our team has previously developed a population pharmacokinetic (popPK) model tailored to Spanish-speaking individuals with opioid use disorder in Puerto Rico (20). This model quantitatively characterizes buprenorphine exposure and predicts steady-state plasma levels under different dosing regimens. Notably, the model suggests that standard daily dosing may be insufficient to maintain therapeutic plasma levels (>3 ng/mL) in a significant proportion of patients, particularly those with altered absorption or metabolism. In contrast, higher total daily doses administered in divided dosing schedules (e.g., 8 mg TID or 16 mg BID) were shown to better sustain receptor saturation and minimize trough concentrations.

These findings support the use of the ODAS as a practical and psychometrically sound tool for assessing buprenorphine dose adequacy in Spanish-speaking populations outside of Europe. The scale’s ability to capture multidimensional markers of clinical stability, combined with pharmacokinetic data, highlights the complexity of dose-response relationships in opioid use disorder treatment. Notably, participants classified as inadequately dosed exhibited lower plasma-to-dose ratios despite receiving higher daily doses, suggesting suboptimal exposure efficiency. Divided or asymmetric dosing patterns, commonly observed in this sample, may contribute to these discrepancies. To address this, the integration of a population pharmacokinetic (popPK) model with ODAS-based assessment offers a translational approach for identifying patients who may benefit from optimized dosing schedules. This model may be used to guide individualized dose adjustments, aiming to maintain therapeutic plasma levels and improve treatment outcomes. In the meantime, providers can use ODAS scores in conjunction with assessments of objective withdrawal symptoms and patient-reported dosing behaviors to inform clinical decisions or reinforce buprenorphine adherence education. Ultimately, combining clinical insight with pharmacokinetic guidance represents a promising strategy to enhance treatment precision and effectiveness among Spanish-speaking individuals with opioid use disorder.

## 5 Conclusion

The psychometric evaluation of the ODAS in this Hispanic sample lays the groundwork for its broader application across Spanish-speaking populations in the Americas. As opioid use disorder continues to disproportionately affect underserved and linguistically diverse communities in the United States and Latin America, the availability of culturally responsive and clinically validated tools to assess treatment adequacy is increasingly critical (21, 22). The ODAS, with its multidimensional structure and adaptable interview format, offers a valuable resource to support evidence-based decision-making, guide individualized buprenorphine dose adjustments, and promote more equitable treatment outcomes in diverse care settings. The present study demonstrates that the ODAS shows acceptable psychometric properties in a non-European Hispanic population, consistent with results from the original Spanish validation and subsequent European studies. Similar to findings in Spanish and German cohorts, we observed good internal consistency and evidence of construct validity, supporting the robustness of the ODAS framework across diverse cultural contexts. However, important differences also emerged. In contrast to European samples, where higher proportions of patients achieved and sustained dose adequacy, our data reflect lower frequencies of dose adequacy, partially explained by features that are common in this population but rarely reported in European cohorts; frequent divided dosing practices. These contextual differences underscore that while the ODAS can reliably capture dose adequacy across populations, interpretation of scores must consider local patterns of treatment delivery and patient behavior.

Clinically, this work highlights the importance of validating measurement tools beyond their original populations. For non-European Hispanic groups, particularly those in high-risk communities with variable treatment adherence, the ODAS offers a structured method for assessing dose adequacy that accounts for the realities of practice. Incorporating the ODAS into care in such settings may support more precise dose adjustments and improved treatment retention. By documenting both consistencies and differences relative to European validation studies, our findings contribute to a more generalizable evidence base for the ODAS and emphasize the need to tailor implementation strategies to cultural and clinical contexts.

## Data Availability

The raw data supporting the conclusions of this article will be made available by the authors, without undue reservation.
